# Sneddon syndrome: a comprehensive clinical review of 53 patients

**DOI:** 10.1007/s00415-021-10407-x

**Published:** 2021-01-29

**Authors:** N. L. P. Starmans, M. R. van Dijk, L. J. Kappelle, C. J. M. Frijns

**Affiliations:** 1 Department of Neurology and Neurosurgery, University Medical Centre, Room G03.232, PO Box 85500, 3508 GA Utrecht, The Netherlands; 2 Department of Pathology, University Medical Centre Utrecht, Utrecht, The Netherlands

**Keywords:** Livedo reticularis, Stroke, Sneddon syndrome, Antiphospholipid antibodies, Risk factors

## Abstract

**Background:**

The presence of livedo reticularis in patients with ischaemic stroke is associated with Sneddon syndrome (SS). Our objective was to present the clinical features of SS patients and to assess the role of antiphospholipid antibodies (APL).

**Methods:**

Consecutive patients, diagnosed with SS between 1996 and 2017, were retrospectively reviewed for their demographic, neurological, dermatological, cardiac and extracerebral vascular features. Diagnosis of SS was made only if other causes of stroke were excluded. Patients with and without APL were included and compared for their clinical features.

**Results:**

Fifty-three patients (79% female) were included, of whom 14 patients were APL-positive. Median age at diagnosis was 40 years. Approximately 60% of the patients had ≥ 3 cardiovascular risk factors. There were 129 previous vascular events (66 ischaemic strokes, 62 TIAs and 1 amaurosis fugax) during a median period of 2 years between the first event and diagnosis of SS. Skin biopsy was positive for SS in 29 patients (67%), mostly showing a thickened vessel wall with neovascularization in the deep dermis. After a median follow-up of 28 months, 4 patients, either on antiplatelet or oral anticoagulation therapy, had a recurrent stroke. There were few statistically significant differences between APL-negative and APL-positive patients, including the number of vascular events before diagnosis.

**Conclusions:**

SS predominantly affects young women with a relatively large number of cardiovascular risk factors. Clinical features of SS are comparable across different studies. We found no differences in the main clinical features between APL-positive and APL-negative patients.

## Introduction

After exclusion of more common causes of stroke, livedo reticularis may be a clue to the aetiology of ischaemic stroke, leading to a diagnosis of Sneddon syndrome (SS) [1–4]. It is a rare disorder, which predominantly affects young women [2, 5]. SS may involve various other organ systems, including the heart, blood vessels and kidneys, and may also cause thrombotic and obstetric complications [2–8]. Common neurological manifestations are ischaemic or haemorrhagic stroke, transient ischaemic attack (TIA), cognitive impairment, headache and epilepsy [2–4]. There are no formal clinical or pathological criteria for the diagnosis of definite or probable SS.

SS is considered to be a non-inflammatory thrombotic vasculopathy that involves the small- and medium-sized arteries of the skin [2]. Various theories about the aetiology of this disorder have been proposed, but no consensus has been reached [2]. At present, 2 groups of patients can be differentiated: those with and those without antiphospholipid antibodies (APL). APL-positive patients can also be diagnosed with the antiphospholipid syndrome (APS), which has overlapping features with SS [9]. However, the presence of livedo reticularis is not required for a diagnosis of APS [9].

The objective of this study was (1) to present the clinical features of a large group of patients with SS and (2) to compare these features in patients with or without APL.

## Patients and methods

### Study population

This retrospective study analysed consecutive patients who were examined between 1996 and 2017 at the Cerebrovascular Clinic of the Department of Neurology and Neurosurgery of the University Medical Centre Utrecht, which is a tertiary referral centre in The Netherlands. All patients were diagnosed with SS with or without APL. SS was defined by the presence of a permanent livedo reticularis involving the legs, trunk and/or buttocks and at least 1 clinically apparent or silent stroke for which no other cause was found despite extensive ancillary investigations [3, 4]. Patients were defined as APL-positive if lupus anticoagulant (LAC), anticardiolipin antibodies (ACA) or anti-β2 glycoprotein-1 antibodies (anti-β2GP1 antibodies) were found at least 2 times with an interval of at least 12 weeks [3, 4].

### Data collection

Data were retrospectively obtained from the patient files and imaging databases.

Baseline characteristics included sex, age at the first clinically apparent stroke and time of diagnosis of SS. Cardiovascular risk factors included hypertension (repeated systolic blood pressure > 140 mmHg or diastolic blood pressure > 90 mmHg), smoking (current or former), diabetes mellitus (repeated fasting glucose ≥ 7.0 mmol/l), overweight (body mass index > 25 kg/m^2^ or based upon expert opinion of the treating physician), dyslipidaemia (total cholesterol > 6.5 mmol/l or LDL cholesterol > 3.5 mmol/l), hyperhomocysteinaemia (> 14.0 µmol/l), atrial fibrillation, oestrogen-containing medication and a positive family history (at least 1 first-degree relative with cardiovascular disease before age 60).

Events were subdivided into ischaemic stroke, haemorrhagic stroke, TIA or amaurosis fugax. TIA was defined as an acute transient episode of neurological deficit caused by focal brain ischaemia without acute infarction on brain imaging or without brain imaging performed [10]. Cognitive impairment was rated as absent, mild (subjective cognitive complaints without functional impairment), moderate (objective cognitive problems in 1 domain with mild functional impairment) or dementia (objective cognitive problems in at least 2 domains with clear functional impairment) [3]. All patients or their partners were asked about cognitive complaints. Patients with cognitive complaints underwent MMSE only (*n* = 2), a short neuropsychological screening battery (*n* = 6) or a full neuropsychological examination (*n* = 2), or were not tested yet (*n* = 3). Headaches were classified as migraine with aura, migraine without aura, chronic tension-type headache or unclassified headache [11].

The presence of livedo reticularis was recorded in 5 locations: face, arms, legs, trunk and buttocks. Skin biopsy results were documented. A standardized skin biopsy was defined as 3 biopsies that were taken from the white centre of the livedo and reached at least until the dermis–subcutis border [12]. Skin biopsies that did not fulfil these criteria were considered unstandardized.

Cardiac examination was performed by a transthoracic or transoesophageal echocardiogram. Libman–Sacks endocarditis, leaflet thickening, valvulopathy or other abnormalities were recorded.

We also collected information about previous arterial thrombosis, venous thrombosis, pulmonary embolism or spontaneous abortion after 10 gestational weeks.

Computed tomography (CT) or magnetic resonance imaging (MRI) was performed at diagnosis and at the end of the follow-up. All scans were rereviewed by one of the authors (NS) and in case of doubt also by a second author (CF). Cerebral infarcts were subdivided into large corticosubcortical infarcts (involving > 2 gyri), small corticosubcortical infarcts (involving ≤ 2 gyri), subcortical infarcts (located subcortically and ≥ 15 mm), lacunar infarcts (< 15 mm) and cerebellar infarcts. Infarcts were localised according to their arterial territories. White matter abnormalities were assessed with the Fazekas scale [13]. Stenosis or occlusion of large extra- and intracranial arteries was identified by CT or MR-angiography. Intracranial haemorrhages were subdivided into lobar or deep haemorrhages.

Laboratory investigations included blood cell count, fasting glucose, HbA1c, fasting total cholesterol, fasting LDL cholesterol, fasting triglycerides, creatinine, estimated glomerular filtration rate (eGFR), proteinuria, APL, antinuclear antibodies (ANA), anti-double-stranded DNA antibodies (anti-ds DNA antibodies), homocysteine, complement levels and several thrombophilia tests. The presence of APL was determined according to international guidelines by a clotting assay based on the activated partial thromboplastin time in case of LAC and by ELISA immunoassays in case of IgM and IgG ACA or anti-β2GP1 antibodies [14, 15]. Due to the long recruitment time in which we included patients, the test kits differed over time, but the type of laboratory test remained the same. Cerebrospinal fluid analyses included cell count, total protein and glucose.

Information about the use of antithrombotic medication included type and indication of the antiplatelet or oral anticoagulation therapy before the first stroke, after the first stroke, after the diagnosis of SS was made and after a recurrent stroke.

Follow-up data included follow-up duration, mortality, disability score (retrospectively evaluated by the modified Rankin Scale (mRS) from the patient files) and stroke recurrence [16].

### Data analysis

All analyses were performed using SPSS version 25. Data are presented as frequencies with percentages of total or median with range. Due to the small study population, nonparametric tests were used to compare patients with or without APL. Fisher’s exact tests were used to compare dichotomous variables and Mann–Whitney *U* tests were used to compare continuous variables. A *p* value of < 0.05 was considered statistically significant.

## Results

### Baseline characteristics

We identified 53 patients with a diagnosis of SS. The majority of the patients were female (Table 1). Median age at the first clinically apparent stroke or TIA was 36 years and the median age at the time of diagnosis was 40 years. Approximately 45% of the patients had ≥ 4 cardiovascular risk factors. One patient had no cardiovascular risk factors.

**Table 1:** Baseline characteristics of the patients at diagnosis

Characteristic	Value (*N* = 53)
	*N* (%) Median (range)
Demographic data	
Female	42 (79)
Age at onset of first vascular event (years)	36.0 (16.0–55.0)
Age at diagnosis (years)	40.0 (21.0–63.0)
Cardiovascular risk factors	
Hypertension	32 (60)
Smoking	42 (79)
Diabetes mellitus	0 (0)
Overweight	20 (39)
Dyslipidaemia	23 (43)
Hyperhomocysteinaemia^a^	17 (43)
Atrial fibrillation	0 (0)
Usage of oestrogen containing medication^a^	11 (24)
Positive family history of cardiovascular disorders	18 (35)
≥ 3 Cardiovascular risk factors	34 (64)
≥ 4 Cardiovascular risk factors	24 (45)

^a^Data on the presence of hyperhomocysteinaemia and usage of oestrogen-containing medication was missing in 13 and 7 patients, respectively

### Extracranial vascular events

There were 10 patients (19%) who also had suffered from extracerebral vascular events consisting of 3 episodes of arterial thrombosis, 5 episodes of deep venous thrombosis in a leg, 3 pulmonary embolisms and 6 spontaneous abortions after 10 gestational weeks.

### Neurological findings

In total, 129 previous events (66 ischaemic strokes, 62 TIAs and 1 amaurosis fugax) had occurred during a median period of 2 years between the first event and the diagnosis of SS. The median number of events before diagnosis was 2. Cognitive impairment was diagnosed in 24% and headaches were reported by 53% of the patients (Table 2).

**Table 2:** Overview of neurological signs and symptoms at diagnosis

Characteristic	Value (*N* = 53)
	*N* (%)
Neurological index event	
Type of event	
Ischaemic stroke	35 (66)
TIA	10 (19)
Amaurosis fugax	0 (0)
Haemorrhagic stroke	0 (0)
Cognitive impairment	2 (4)
Silent stroke	6 (11)
Symptoms of event^a^	
Motor deficit	29 (66)
Sensory deficit	17 (39)
Aphasia	14 (32)
Dysarthria	9 (21)
Hemianopia	5 (11)
Cortical blindness	1 (2)
Diplopia	2 (5)
Central facial palsy	10 (23)
Cerebellar ataxia	3 (7)
Other neurological symptoms	
Cognitive impairment	
None	40 (76)
Mild	6 (11)
Moderate	6 (11)
Dementia	1 (2)
Headache	
Any type	28 (53)
Migraine with aura	10 (36)
Migraine without aura	9 (32)
Chronic tension-type headache	7 (25)
Unclassified headache	2 (7)
Epilepsy	
Focal	4 (8)
Generalised	0 (0)

TIA indicates transient ischaemic attack

^a^Totals can exceed 100%, because patients can have multiple symptoms per stroke

### Dermatological findings

The median number of locations of livedo was 3 out of 5 (Table 3). Eight patients (15%) presented with livedo in only 1 location. In 5 out of 6 biopsies in these patients, the results supported the diagnosis of SS. Furthermore, Raynaud’s phenomenon was present in the medical history or during the physical examination in 13 patients.

**Table 3:** Overview of dermatological signs and symptoms at diagnosis

Characteristic	Value (*N* = 53)
	*N* (%)
Location of livedo reticularis	
Face	7 (14)
Arms	40 (77)
Legs	52 (98)
Trunk	34 (65)
Buttocks	8 (15)
Trunk and/or buttocks	35 (67)
Skin biopsy performed	43 (81)
Skin biopsy standardized^a^	20 (47)
Sneddon syndrome-related abnormalities	29 (67)
Type of abnormalities^b^	
Thickened vessel wall	20 (69)
Neovascularization	27 (93)
Occluded vessels	10 (35)
Inflammatory cells surrounding vessels	6 (21)
Location of abnormalities	
Superficial dermis	1 (4)
Deep dermis	20 (71)
Total dermis	7 (25)

^a^Unstandardized procedures included less than 3 biopsies (*n* = 15), biopsies not deep enough (*n* = 7) or not taken from the white centre of the livedo (*n* = 3)

^b^Totals can exceed 100%, because skin biopsies can show multiple abnormalities

Skin biopsy results were positive for SS in 29 patients (67%) (Table 3). The most frequently encountered abnormalities were a thickened vessel wall with neovascularization in the deep dermis. There were no signs of vasculitis. Occluded vessels were more frequently found in the standardized (50%) than in the unstandardized skin biopsies (20%) (*p* = 0.13).

### Cardiac examination

Forty-nine patients had a cardiac examination by either transthoracic (*n* = 18), transoesophageal (*n* = 28) or unknown type of echocardiogram. Examination revealed abnormalities in 23 patients (47%). Libman–Sacks endocarditis was present in 2 patients (2 mitral valves), leaflet thickening in 4 patients (3 aortic and 1 mitral valve) and valve regurgitation in 7 patients (4 aortic, 2 mitral and 1 tricuspid valve). No patients had valvular stenosis or mixed valvular disease. Left ventricular hypertrophy and segmental wall motion abnormalities were reported in 1 patient. Other abnormalities included an atrial septal defect (*n* = 3), patent foramen ovale (*n* = 3) and bicuspid aortic valve (*n* = 5).

### Radiological findings

At diagnosis, a MRI scan was performed in 48 patients and a CT scan was performed in 4 patients. The scan of 1 patient reporting multiple lacunar infarcts could not be retrieved and was, therefore, excluded from the analyses. There were 179 cerebral infarcts, resulting in a median number of 3 infarcts per patient (Table 4). Approximately 70% of the patients had infarcts in multiple arterial territories.

**Table 4:** Radiological findings at diagnosis

Characteristic	Value (*N* = 53)
	*N* (%)
Total number of	
Cerebral infarcts	179 (100)
Large corticosubcortical	65 (36)
Small corticosubcortical	61 (34)
Subcortical	8 (5)
Lacunar	16 (9)
Cerebellar	29 (16)
Intracerebral haemorrhages	0 (0)
Arterial territories	
Anterior cerebral artery	10 (19)
Middle cerebral artery	47 (90)
Posterior cerebral artery	23 (44)
Cerebellar arteries	18 (35)
Multiple arterial territories	37 (71)
White matter abnormalities	
Presence	40 (83)
Severity	
Mild	38 (95)
Moderate	2 (5)
Extensive	0 (0)
Location	
Supratentorial	31 (78)
Infratentorial	0 (0)
Both	9 (23)
Stenosis or occlusion	
Intracranial	10 (23)
Extracranial	3 (7)

A brain scan at the end of the follow-up was available in 26 patients and was compared with the scan at diagnosis. The number of infarcts on the follow-up scans was unchanged in 18 patients. The other scans showed an increase of the number of infarcts (*n* = 5), an increase of white matter abnormalities (*n* = 3) or more stenosed or occluded vessels (*n* = 2).

### Laboratory data

Standard laboratory tests were normal, except for a decreased eGFR in 6 patients. Homocysteine was elevated in 17 out of 40 patients (median of 21.3 µmol/l, range 14.4–73.0). There were 14 out of 53 APL-positive patients. Seven patients had LAC, 3 patients had IgM ACA, 7 patients had IgG ACA, 1 patient had IgM anti-β2GP1 antibodies and 4 patients had IgG anti-β2GP1 antibodies. Three patients were APL triple positive. ANA were present in 14 out of 44 patients. Anti-ds DNA antibodies were present in only 1 patient, who did not meet the diagnostic criteria for systemic lupus erythematosus. Thrombophilia tests were performed in 26 patients and revealed 1 protein C deficiency, 2 protein S deficiencies, 2 heterozygotes and 1 homozygote factor V Leiden mutation and 1 prothrombin mutation. Cerebrospinal fluid data showed isolated elevated protein levels in 10 out of 20 patients with median protein levels of 0.72 g/l. Cell counts were normal in all patients.

### Antithrombotic therapy data

Four patients used antithrombotic agents before the first stroke, because of heart valve disease, recurrent spontaneous abortions, recurrent deep venous thrombosis or possible Büerger’s disease. After the first stroke, 49 patients received antiplatelet therapy and 3 patients received oral anticoagulation therapy. After SS was diagnosed, all patients received antithrombotic agents (antiplatelet therapy in 38 and oral anticoagulation therapy in 15).

### Follow-up data

Follow-up was available in 31 patients (59%). Median follow-up duration since diagnosis was 28.0 months (range = 7.0–197.0). One patient died 58 months after diagnosis due to pneumonia and another patient died 23 months after diagnosis due to sepsis and multi-organ failure. Median mRS at the end of the follow-up was 2. There were 7 patients (26%) with a mRS ≥ 3.

Four patients (13%) had a recurrent stroke during follow-up, including 4 ischaemic strokes and 1 silent stroke (Table 5). Strokes recurred in 2 out of 20 patients on antiplatelet therapy during 77.6 patient years and in 2 out of 11 patients on oral anticoagulation therapy during 66.0 patient years. The median time to the first recurrent stroke was 26.5 months in the patients on antiplatelet therapy and 46.0 months in the patients on oral anticoagulation therapy.

**Table 5:** Number of patients with stroke recurrence during follow-up

Characteristic	Value (*N* = 31)
	*N* (%)
Ischaemic stroke	3 (10)
TIA	7 (23)
Amaurosis fugax	2 (7)
Haemorrhagic stroke	0 (0)
Silent	1 (4)

TIA indicates transient ischaemic attack

### Differences between APL-negative and APL-positive patients

The following features differed significantly between the APL-negative and APL-positive patients: median number of cerebral vascular events before diagnosis (2.0 versus 1.0 respectively, *p* = 0.03), the presence of other non-valvular cardiac abnormalities, such as an atrial septal defect, (31% versus 0%, respectively, *p* = 0.02), presence of deep venous thrombosis (0% versus 21%, respectively, *p* = 0.02) and presence of ANA (21% versus 70% respectively, *p* = 0.01). The other features did not differ significantly. Epileptic seizures and recurrent strokes were only seen in APL-negative patients (*p* = 0.56 and *p* = 0.28, respectively). Libman–Sacks endocarditis was only seen in APL-positive patients (*p* = 0.07).

## Discussion

In this study we presented the clinical features of 53 consecutive patients, who were diagnosed with SS, with or without APL. An important finding was the strikingly high prevalence of vascular risk factors in this relatively young and mainly female population. Most patients had cerebral infarcts in more than one territory. There were only a few non-specific differences between APL-positive and APL-negative patients.

The prevalence of cardiovascular risk factors in this population is much higher when compared to the general population in The Netherlands [17]. A large prospective cohort study reported a prevalence of hypertension of 22.5% and smoking of 21.5% in adults between 18 and 65 years old [17]. Our findings are in line with those of a single-centre, large study in 53 APL-negative SS patients that reported at least 2 cardiovascular risk factors in 64% of the patients [3]. Our high number of cardiovascular risk factors can be partly explained by the number of cardiovascular risk factors that we reviewed, since we also included hyperhomocysteinaemia, use of oestrogen-containing medication and a positive family history of cardiovascular disorders. A previous study of 27 APL-negative and 19 APL-positive SS patients also reviewed these cardiovascular risk factors and found a similar number of patients on oestrogen-containing medication or with a positive family history, but a lower number of patients with hyperhomocysteinaemia [4].

The relatively large prevalence of APL is probably caused by patient selection, as APL can be part of the SS. Similar numbers of APL-positive patients were found by another large cohort [4]. These numbers cannot be directly compared to the general population, or even a general stroke population. It is unclear if these APL have a role in the pathophysiology of SS. Therefore, they could be an epiphenomenon, but they could also have a role in creating a prothrombotic state.

APL-negative patients with SS more often had multiple strokes before the diagnosis was made than APL-positive patients. This has also been found in a previous study [3]. Possibly, the diagnosis of SS is easier, and therefore, earlier, to make in APL-positive than in APL-negative patients. Contrastingly, there were no haemorrhagic strokes, although these have been reported in some series and case reports [3, 18, 19]. The largest series reported 7 haemorrhagic strokes in 53 patients and also highlighted that 20% of the infarcts were located in the cerebellum [3]. Our study confirmed the frequent occurrence of small cerebellar infarcts. Furthermore, about a quarter of our patients showed cognitive impairment. This prevalence was not as high as in previously published studies, possibly because we did not perform extensive neuropsychological assessments in all patients [3, 4].

Earlier studies only included patients if they had widespread permanent livedo reticularis involving the trunk and/or buttocks assessed by an expert senior dermatologist [3, 4]. However, in some patients, livedo reticularis may be less widespread, but still be relevant for the diagnosis of SS. This was demonstrated by the patients with limited livedo in our study in whom skin biopsy was positive. Livedo reticularis is quite a common finding amongst young women and may be found by chance. Therefore, we aim to perform skin biopsies in the majority of patients to support the clinical diagnosis of SS. Skin biopsy results were in support of the diagnosis in 67% of the patients. In previous studies, skin biopsies were performed less often and were found positive in less than 30% of the patients [3, 4]. The sensitivity of the biopsies may vary according to the method of performance and may increase to 80% when 3 deep 4-mm punch biopsies of the white centre of the livedo are performed [12]. In addition, pathological criteria may differ as there are no established pathological criteria for the diagnosis of SS. For example, one study reported occluded vessels in all biopsies, whereas we mostly found a thickened vessel wall and neovascularization in support of the clinical diagnosis [4]. Robust pathological criteria may greatly aid in making this diagnosis, and pathological research may help in elucidating the pathophysiology.

During follow-up, 4 patients with SS had recurrent strokes despite antiplatelet or oral anticoagulation therapy. Previous studies showed similar recurrences in patients on either antiplatelet or oral anticoagulation therapy [3, 4, 20].

We found some differences between the APL-negative and APL-positive patients, which may in part be associated with APS, such as more frequent venous thrombosis and positive ANA. Only 1 other study compared the clinical features of APL-negative and APL-positive SS patients [4]. They reported that epileptic seizures, mitral regurgitation and thrombocytopaenia were significantly more frequent in APL-positive than in APL-negative patients [4]. However, because of the retrospective nature and the small number of patients in both studies, relevant clinical differences can be missed. On the other hand, the described differences between the 2 groups may be because of selection bias or by chance, since not all patients were tested and multiple comparisons were made.

Our study has several strengths. First, we gave a comprehensive overview of the different clinical features of SS in a relatively large number of patients. Second, skin biopsies were performed in the majority of the patients, providing more insight into the pathological skin findings. Third, we reviewed all available brain scans according to predefined criteria, so we were able to report all cerebral infarcts and corresponding arterial territories. However, due to the lack of a reference standard, we cannot completely exclude that for example cardiovascular risk factors or cardiac abnormalities caused the ischaemic strokes, and that some patients, in fact, do not suffer from SS. The presence of livedo could then be considered as a coincidence. In addition, the retrospective nature of the study led to some missing data and unclear definitions.

Taking both the limited literature and our experience into account, we treat patients with antiplatelet therapy in the absence of a cardioembolic source and switch early to oral anticoagulation therapy in case of any recurrent TIA or ischaemic stroke. In addition, a meticulous search and treatment of all cardiovascular risk factors is warranted. Radiological follow-up to screen for new silent infarcts may be considered.

Despite the fact that the knowledge of SS is expanding, questions about aetiology, diagnosis and treatment remain unanswered. At present, robust clinical and pathological criteria for the diagnosis of SS are lacking. Larger, prospective and multicentre studies could provide some answers on the pathophysiology, the role of skin biopsies in making the diagnosis, optimal treatment strategies and the prognosis of these patients.

## Conclusion

SS is a disease that predominantly affects young women. Patients typically present with multiple cerebral infarcts, a relatively large number of cardiovascular risk factors and involvement of various other organs, such as the heart. There were no differences in the main clinical features of cerebrovascular disease, livedo reticularis and cardiovascular risk factors between the APL-positive and APL-negative patients. There were few differences in associated signs and symptoms. Treatment to prevent recurrent strokes and limit disability should include antithrombotic therapy and elimination or treatment of cardiovascular risk factors.

## Data Availability

To minimise the possibility of unintentionally sharing information that can be used to re-identify private information, a subset of the anonymized data is available from the corresponding author upon reasonable request.
